# Myosin Va but Not nNOSα is Significantly Reduced in Jejunal Musculomotor Nerve Terminals in Diabetes Mellitus

**DOI:** 10.3389/fmed.2014.00017

**Published:** 2014-06-20

**Authors:** Arun Chaudhury, Marcilio Hubner De Miranda-Neto, Renata Virginia Fernandes Pereira, Jacqueline Nelisis Zanoni

**Affiliations:** ^1^Department of Surgery, Brigham and Women’s Hospital, Harvard Medical School and VA Boston HealthCare System, West Roxbury, MA, USA; ^2^Department of Morphological Sciences, Universidade Estadual de Maringá, Maringá, Brazil

**Keywords:** axonal transport, myosin Va, nNOS, diabetes, neuropathy

## Abstract

Nitric oxide (NO) mediated slow inhibitory junction potential and mechanical relaxation after electrical field stimulation (EFS) is impaired in diabetes mellitus. Externally added NO donor restore nitrergic function, indicating that this reduction result from diminution of NO synthesis within the pre-junctional nerve terminals. The present study aimed to investigate two specific aims that may potentially provide pathophysiological insights into diabetic nitrergic neuropathy. Specifically, alteration in nNOSα contents within jejunal nerve terminals and a local subcortical transporter myosin Va was tested 16 weeks after induction of diabetes by low dose streptozotocin (STZ) in male Wistar rats. The results show that diabetic rats, in contrast to vehicle treated animals, have: (a) nearly absent myosin Va expression in nerve terminals of axons innervating smooth muscles and (b) significant decrease of myosin Va in neuronal soma of myenteric plexus. In contrast, nNOSα staining in diabetic jejunum neuromuscular strips showed near intact expression in neuronal cell bodies. The space occupancy of nitrergic nerve fibers was comparable between groups. Normal concentration of nNOSα was visualized within a majority of nitrergic terminals in diabetes, suggesting intact axonal transport of nNOSα to distant nerve terminals. These results reveal the dissociation between presences of nNOSα in the nerve terminals but deficiency of its transporter myosin Va in the jejunum of diabetic rats. This significant observation of reduced motor protein myosin Va within jejunal nerve terminals may potentially explain impairment of pre-junctional NO synthesis during EFS of diabetic gut neuromuscular strips despite presence of the nitrergic synthetic enzyme nNOSα.

## Introduction

Slow inhibitory junction potentials (sIJP) are recorded using impaled electrodes within gastrointestinal smooth muscles in response to nitric oxide (NO) released from pre-junctional nerve terminals after electrical field stimulation (EFS). Independent studies have confirmed impairment of sIJP in muscle samples obtained from all portions of the gastrointestinal tract of animals with pharmacologically induced or genetically acquired diabetes mellitus (1–3). Mechanical studies have confirmed diminution of EFS-mediated nitrergic relaxations in diabetic enteric tissues like ileum and mid-colon (3–7). Some of these studies have also demonstrated restoration of nitrergic mediated responses in diabetic tissues with externally added NO donor to the organ bath (4–6), indicating that the diminution of nitrergic function in diabetic gut tissues resulted primarily from reduction or inhibition of NO synthesis within the pre-junctional nerve terminals.

Controversy exists in the reports regarding the content of neuronal nitric oxide synthase (nNOS) within the enteric nerve terminals in diabetic gut tissues. Limited data showing only a single low power microscopic field have been used to report reduced number of nitrergic axons traversing the neuromuscular wall in streptozotocin (STZ) induced diabetes (8). Other studies have reported reduced dimer/monomer ratio of nNOS (9) or increase in total nNOS levels in diabetes (9, 10). However, the nNOS blots were run with extracts obtained from whole gut tissues, thus precluding specific information about nNOS contents within the nerve terminals *per se*. Studies have also reported normal nNOS enzymatic activity of diabetic whole gut extracts assayed *in vitro* (11). However, none of these studies provide unequivocal evidence about nNOS contents within the nerve terminals, the site of inhibitory enteric neuromuscular nitrergic neurotransmission.

Recently, evidence has been provided that mere presence of nNOS within nerve terminals is not adequate for pre-junctional NO synthesis (12–14). The regulation of nNOS within the nerve varicosities require multiple allosteric interactions, most notably, its positioning at PDZ-rich active zones that allow interfacing of water soluble nNOSα with membrane-bound palmitoyl-PSD95 (14, 15). Intriguingly, this binding of nNOSα is not stochastic and dependent on a Brownian kind of diffusion but rather relies on specific molecular interactions involving motor proteins like myosin Va that have the ability to deliver nNOSα to membrane-binding sites (12). Using a mouse model of myosin Va mutation, the dilute DBA/2J mice, it was shown that *in vitro* NO synthesis of enteric synaptosomes and NO-mediated sIJP and l-NAME sensitive mechanical relaxations were impaired in gastric tissues of dilute mice (12, 14).

The purpose of the present study was to investigate two specific aims that may potentially provide pathophysiological insights into diabetic enteric nitrergic neuropathy: (a) is there any alteration in nNOS contents within enteric nerve terminals in diabetes (b) is there any alteration in myosin Va contents within enteric nerve terminals in diabetes. The first question was pursued to obtain unambiguous evidence about the state of nitrergic nerve terminals in diabetes at a fixed time point after diabetes induction. Based on preliminary evidence that local intravaricosity transport of nNOSα by myosin Va motor protein is important for efficient NO synthesis during neurotransmission (12, 13), we hypothesized that deficiency of myosin Va may contribute to impaired nitrergic neurotransmission in diabetes.

## Materials and Methods

### Induction of diabetes mellitus

All experimental procedures were conducted with approval from IACUC Committee of VA Boston HealthCare System (VABHS) and Committee of Ethics in Animal Experimentation from the Universidade Estadual de Maringa’. Male Wistar rats (*Rattus norvegicus*) from the Central Biotery of Universidade Estadual de Maringa’ were utilized for the present investigation. Ninety-day-old rats were maintained in separate cages for 4 months prior to experimentation. After a 14-h fast, STZ (35 mg/kg body weight; Sigma, St. Louis, MO, USA) dissolved in freshly prepared 10 mM citrate buffer (pH 4.5) was intravenously injected. Animals with persistent plasma glucose >250 mg% were used for the study as a model of diabetes mellitus. Normoglycemic vehicle treated animals were used as controls. Jejunum were obtained after 4 months of diabetes induction. Details of tissue procurement and euthanasia procedures have been described earlier (16).

### Staining of jejunal whole mounts for myosin Va and nNOSα

Details of tissue fixation and staining were performed as per protocols described earlier (16). Jejunum was used as representative of gut neuromuscular strip. A polyclonal antibody raised against chicken brain myosin Va (17) and extensively characterized previously was used for myosin Va immunostaining (1 in 750 primary, prepared at University of São Paulo, Ribeirão Preto-campus). Tissue reaction was performed by incubating with hydrogen peroxide and diaminobenzidine (DAB). Omission of the primary antibody did not show any reaction product after labeling. nNOSα immunostaining was performed with a commercial antibody developed against epitope N_2–300_ (H-299, 1 in 500, Santa Cruz Biotechnology) with appropriate negative control. Avidin–biotin histochemistry and Alexa Fluor fluorescent images were separately obtained to visualize nNOSα immunostaining. Uniform staining protocols were used.

### Light microscopic imaging of whole mounts and acquisition of images

Digitized images were obtained with Zeiss Axioskop Plus light microscope at 200-fold magnification and stored for offline analyses. Same scaled images were used for comparative analyses, though digitally zoomed images may have been shown as representative figures for better depiction of the projected features.

### Pseudocoloring of images for creating contrast and better visualization of neuromuscular structures

Some image profiles were pseudocolored to create better contrast for visualization of neuromuscular apparatus. Images were opened in ImageJ (NIH freeware)^1^ and converted to 16- or 32-bits prior to choosing a pseudocolor (sepia, magenta hot, blue orange icb, and glasbey) from the lookup table (LUT) for creating efficient contrast.

### Quantitation of space occupancy of nitrergic varicosities

Space occupancy of nitrergic varicosities was obtained using a simple derivative of graph theory. Graph theory relies on connectivity patterns based on number of vertices and edges and maybe expressed as: G = (*V*, *E*) (18, 19). The nerve varicosities may be inferred as the vertices (*V*) and the axonal processes (primary, as well as branches) to be edges (*E*). Based on the argument that cable length and sites of neurotransmission (junctions or varicosities) are critical for determining efficiency of neurotransmission (20), obtaining quantitative information on spatial densities will be a more reliable marker of neurites’ arborization rather than random visualization of a snapshot of a whole mount microscopic field. Quantizing the pixel intensities of varicosities and the processes provide a semiquantitative approach toward estimating whether any changes occurred in the spatial density of nerve terminals after diabetes induction. In order to obtain pixel intensities of axonal processes, repeated thresholding was performed in ImageJ to remove background intensity. The auto-threshold function (Huang, intermodes, Li, maximum entropy, and isodata) in ImageJ was used for these analyses. RGB images were converted to 8 or 16-bit prior to thresholding.

### 3D surface modeling to visualize myosin Va musculomotor terminals

The plugin 3D interactive surface plot in ImageJ was used to visualize the stained varicosities and myenteric plexus. Unaltered images were used for these quantitative analyses. The spike height and color hue are representative of immunoexpression intensity. Same parameters of grid size, smoothing, perspective, and lighting were used for all whole mounts analyzed.

### Estimation of nNOSα contents within nitrergic varicosities

Varicosities were carefully outlined with freehand selection tool in ImageJ and area and integrated densities estimated for nNOSα concentrations within varicosities and intensities normalized to area. Axonal processes were carefully traced from the cell body toward the ramification on the muscular wall and followed till the process ended or faded in a different plane. This information was also used to map a static kymograph that provided insights into nNOSα axonal transport in vehicle treated and STZ-induced diabetes. Linear distances between varicosities were measured. Raw images were used for intensity analyses.

### Neuronal process tracing

Free hand tools were used to trace nitrergic neurons on planar unscaled whole mounts in MS Paint. The trace was independently visually verified in NeuronJ^2^ (data not shown) (21). Random selections of neurons were made from independent whole mounts obtained from different rats.

### Statistical analyses

Quantitative data was expressed as mean ± SEM. Two-tailed *t* test was used to compare difference between means of the parametric datasets.

## Results

### Myosin Va immunoreactivity is scant or nearly absent in the neuro-smooth muscle nerve terminals of jejunum

**Figure 1:**
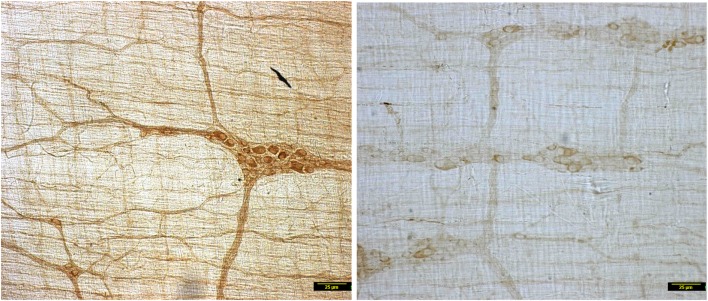
**Low power micrographs from jejunal whole mounts of vehicle treated (left) and streptozotocin induced diabetic rats (right)**. Note the obvious differences in DAB staining intensity of myosin Va between the two panels. Scale bar, 25 μm.

In contrast to the vehicle treated rats, induction of diabetes by STZ resulted in nearly complete loss of myosin Va within the nerve terminals of the neuronal processes ramifying on the muscularis externa (Figure 1). Whole mounts of jejunum stained with myosin Va specific antibody failed to show the brown reaction product of DAB staining that was visualized in the enteric tissues of normal rats (Figure 1). The absence or paucity of myosin Va staining overlying the muscularis externa was apparent during light microscopic imaging.

### Myosin Va immunoreactivity is variably decreased or nearly absent in many neuronal cell bodies of myenteric plexus of jejunum

**Figure 2:**
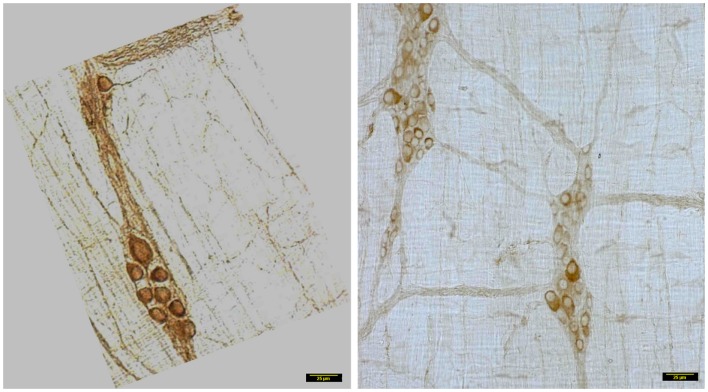
**Myosin Va immunoreactivity extending from the cell body to the processes of Dogiel type 2 neurons emanating from myenteric plexus**. In vehicle treated group (left panel), myosin Va is present in the initial segment, as well as axonal processes extending to the muscle layers. Myosin Va is also present in the interneuronal processes within the ganglion. In contrast, note that in the diabetic rats, despite the presence of secondary plexus linking the ganglia and tertiary extensions of nerve processes into the muscle layers, the brown DAB reaction product representative of myosin Va presences is scant or nearly absent in these processes. Scale bar, 25 μm.

In wild type condition, myosin Va immunoreactivity was strongly present in the cell bodies of all neurons present within the myenteric plexus. The brown reaction product was well visualized and uniformly distributed across the whole cytosol of the neuronal soma. Myosin Va immunoreactivity was also well visualized in the axon initial segment (AIS) of the ganglionic neurons and extended into the processes that link different cells within the ganglion, as well as axonal extensions into the muscularis (Figure 2). There was diffuse staining for some length of the initial parts of the axonal extensions, but thereafter, myosin Va immunoreactivity was seen as regular punctate appearance, indicating probable concentration of myosin Va in the nerve varicosities of the neuro-smooth muscle neuromuscular junctions.

**Figure 3:**
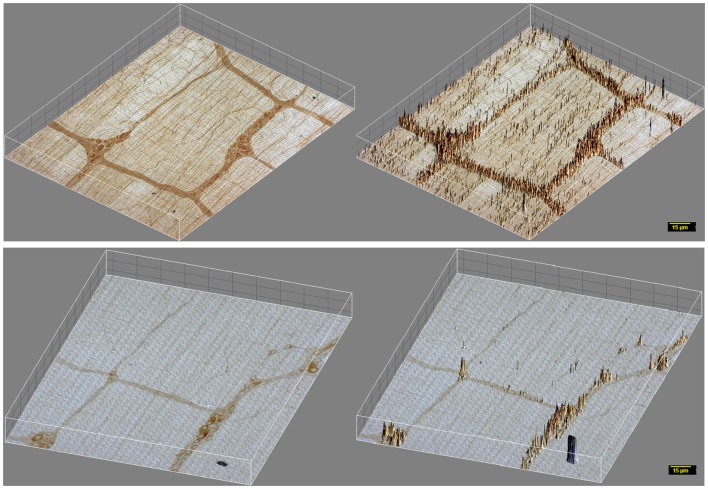
**3D surface plot of DAB staining intensity of myosin Va in WT and diabetic jejunum**. Note that in vehicle treated rats (upper panels), staining spikes present in the muscle segments interposed between the ganglia and plexuses have dense distribution of myosin Va spikes in the nerve terminals, while these spikes within the muscularis are almost absent in diabetic tissues (lower panels). These nerve terminals are the sites of neuro-smooth muscle neurotransmission. Note the lighter hue of the staining spikes within the neuronal cell bodies, representative of reduction in myosin Va genomic expression within the neuronal soma in diabetic tissues in comparison to vehicle treated rats. Scale bar, 15 μm.

**Figure 4:**
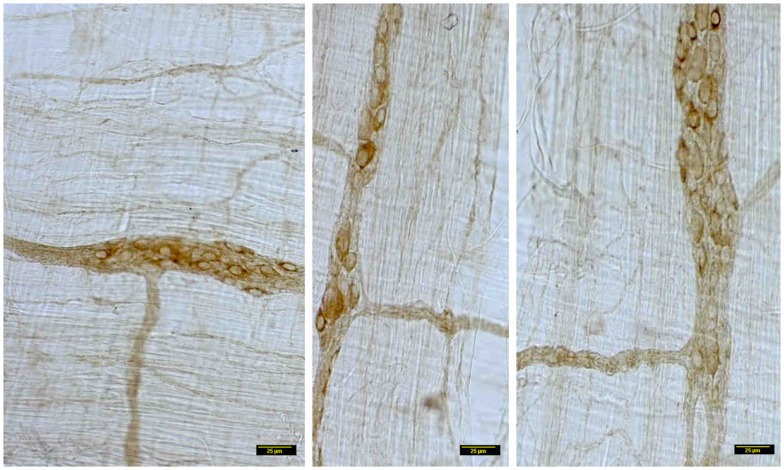
**Details of myosin Va staining of ganglionic neurons and their processes in diabetic jejunum**. Note the heterogeneity of appearance of myosin Va in the cell bodies of the neurons in the myenteric plexus. In the left panel, note the moderate staining in the cell bodies of many neurons. Also note that the secondary plexus running to the left of the image has some brown DAB staining. Some of the neuromuscular varicosities have faint brown staining. In the middle panel, note the enlarged neuronal soma with unhealthy appearing nucleus, possibly indicating a degenerating neuronal cell body. The increase in size may be related to the cellular accumulation of myosin Va, possibly resulting from stasis of myosin Va axonal transport. Also note the absence of brown staining in all of the neuronal processes ramifying through the muscular wall. The right panel recapitulates information represented in the middle panel and in addition, shows a full range of neuronal cytopathology. Note that in the upper pole, two neurons do not have any cytoplasmic staining and the lower neuron is possible degenerative, as evidenced by unusual nuclear appearance. Note very scant or nearly absent staining in the interneuronal processes, secondary plexi as well as tertiary neuromuscular nerve varicosities. Also note that some cells (top right, also one cell body in the right on left panel) show intense dark brown staining. This heterogeneous appearance may result from enhanced myosin Va transcription but may have also resulted from myosin Va aggresome formation. Scale bar, 25 μm.

In contrast, myosin Va immunoreactivity was severely diminished in most neuronal cell bodies in diabetic jejunal whole mounts (Figure 2). In the neuronal cell bodies that demonstrated reduced staining, myosin Va immunoreactivity did not extend even into the AIS (Figure 2). Significantly, no myosin Va immunoreactivity was seen even in the initial portions of the axonal extensions in diabetic jejunum. Myosin Va immunoreactivity was only scantily and rarely present in the nerve varicosities overlying the muscularis in diabetic tissues (Figure 3). Light microscopic imaging reveals the variance of the myosin Va staining and distribution within the ganglion neurons and its arborizations in diabetic tissues (Figure 4). Molecular techniques like western blotting of whole tissue protein extracts would fall short in identifying this significant observation. Because of only sporadic presence of spotty myosin Va-IR within nerve varicosities of diabetic tissues (Figure 3), quantitative myosin Va concentrations within nerve terminals of the neuromuscular junctions were not obtained.

### Unlike myosin Va, nNOSα immunoreactivity is normal in the neuro-smooth muscle nerve terminals of jejunum

**Figure 5:**
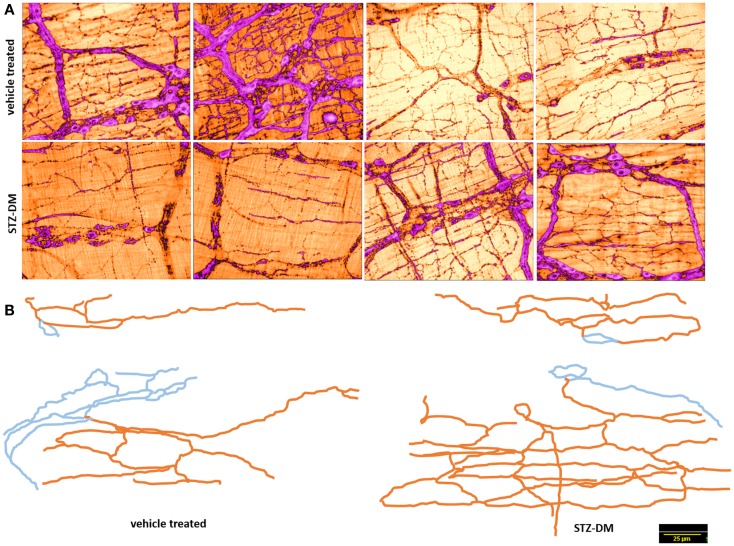
**Normal arborization of nitrergic neuronal processes on muscularis in both vehicle treated and diabetes**. **(A)** shows variance in distribution of the nitrergic processes. **(B)** shows two traced neurons in each group. Note the complex ramifications patterns. Blue indicates cell body and interganglionic neuronal connections. Orange color indicates the neuronal processes extending onto the muscularis. Note that varicosities were not traced. Scale bar, 25 μm.

**Figure 6:**
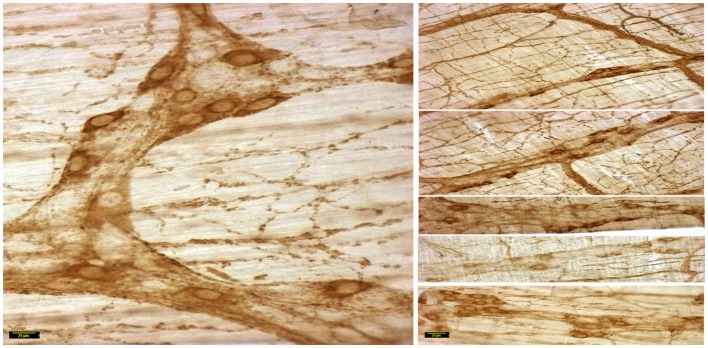
**nNOSα expression in vehicle treated jejunum**. Note the variance of nNOS expression in the neuronal cell bodies in the segments of the right panels. Scale bar representative of each side, 25 μm.

nNOSα immunoreactivity within the nerve terminals in vehicle treated and STZ-induced diabetic jejunal tissues was quantitatively examined. nNOSα was present within the nerve terminals of both vehicle treated and STZ-treated rats (left panels of Figures 5 and 6). Also note that the nerve terminals and nitrergic axons form loop like structures, reminiscent of Kirchoff-type circuits. This distribution may serve to propagate inhibitory nerve signals simultaneously over a segment of muscle, though no electrophysiological evidence or imaging evidence of simultaneous NO production over the loop with a single electrical stimulus has ever been obtained.

### nNOSα immunoreactivity is normal or variably decreased in the neuronal cell bodies of myenteric plexus of jejunum

The variance of nNOSα expression is demonstrated in the right panels of Figures 5 and 6 (vehicle and STZ treatment, respectively). The cellular expression of nNOSα is comparable between vehicle treated normal (Figure 5) and STZ-treated diabetic jejunum (Figure 6). While most of the cells are uniformly stained in the wild type tissues with comparable pixels of expression, the fourth segment in the right panel (Figure 5) shows cell bodies with comparatively low expression of staining. The reason to demonstrate this variance is to stress that mere comparative expression from whole tissue extracts by protein blotting techniques is not optimal to obtain evidence regarding the neuropathology of nNOSα expression. Some of the neuronal cell bodies demonstrate lower levels of expression of nNOSα (Figure 6). However, nNOSα was present in the neuronal processes arising from these cell bodies. Most whole mounts actually demonstrated normal levels of expression of nNOSα in the diabetic tissues. Occasionally, apoptotic neuronal cells with high nucleocytoplasmic ratios and skeletonized outlines were observed (Figure 6, lower segment of the right panel).

### Nitrergic neuronal processes on muscularis externa arborize normally and show no retraction in diabetic jejunum

**Figure 7:**
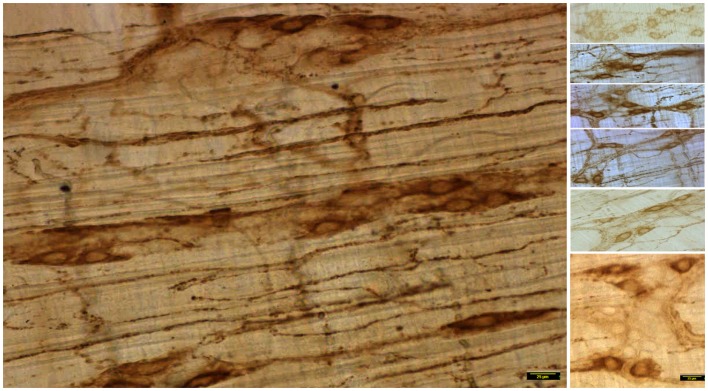
**nNOSα expression in STZ-treated diabetic jejunum**. Note the variance of nNOS expression in the neuronal cell bodies in the segments of the right panels. Note reduction of nNOSα expression in some cell bodies. However, the neuronal processes emanating from these cell bodies show nNOSα expression. Note that the lowest panel shows some degenerating neuronal cell bodies. Scale bar representative of each side, 25 μm.

Observations of the whole mounts show wide variance in the distribution patterns of the processes emanating from the ganglionic neuronal cell bodies and extending to the muscularis externa. Representative tracings indicating this variance is demonstrated in both vehicle treated and diabetic tissues in Figure 7. Several nitrergic neuronal processes were traced and compared between vehicle treated and diabetic tissues. The neuronal arborizations were extensive and cable length comparable between sham treated and diabetic tissues, indicating no retraction of neuronal processes of nitrergic neurons that ramify on the muscularis in diabetes (Figure 7, lower panel). Semiquantitative parameters like the distance of the farthest point on the axonal process from the centroid of the neuronal cell body were used to compare neural arborization. These data were comparable between vehicle and STZ treatment (data not shown).

### nNOSα is normally distributed in the cell bodies of myenteric plexus and extends into the dendrites of Dogiel type 1 neurons and interneuronal processes within ganglia in diabetes

**Figure 8:**
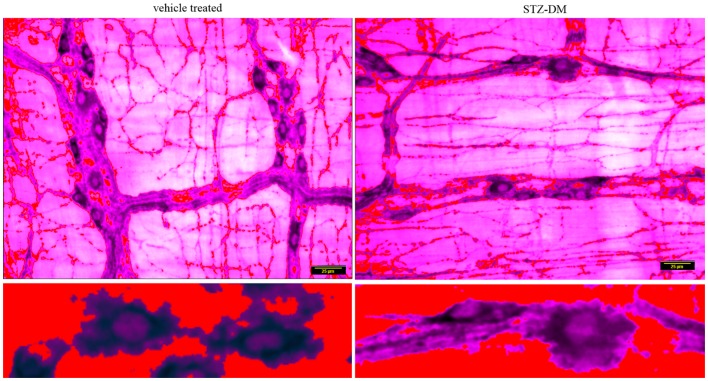
**Normal staining of nNOS within dendritic processes of Dogiel type 1 neurons and interganglionic neuronal processes in both vehicle treated and STZ-induced diabetic jejunum**. The lower panels show a digitally zoomed image showing that nNOS extends into the proximal processes of the nitrergic neurons in both wild type and diabetic jejunal tissues. Renyi entropy type thresholding of pseudocolored images were used to observe the neuronal branch pattern morphology. Scale bar, 25 μm.

nNOSα is present not only in the nerve terminals in diabetes, but also normally distributed within the cell bodies as well as the dendrites of Dogiel type 1 neurons. Images were converted to 16-bits and pseudocolor coded prior to thresholding for optimal visualization of the neuronal cell body and processes. The distribution of nNOSα in wild type and diabetes in the proximal processes were comparable (Figure 8). Interneuronal processes between smooth Dogiel type 2 neurons and axonal extensions into the muscle contained comparable levels of nNOSα in vehicle treated and diabetic tissues. These qualitative observations indicate that at the time period examined, nNOSα axonal and dendritic transport is not impeded despite diabetic condition of the rats. Again, it may be appreciated that the genomic expression of nNOSα is variable and may be slightly diminished in some ganglionic neurons. The intact axonal transport of nNOSα in diabetes is sharply contrasted against the nearly absent axonal transport of myosin Va in diabetes at the specific time point examined.

### Space occupancy of nitrergic varicosities are comparable between vehicle treated and STZ-induced diabetic jejunum

**Figure 9:**
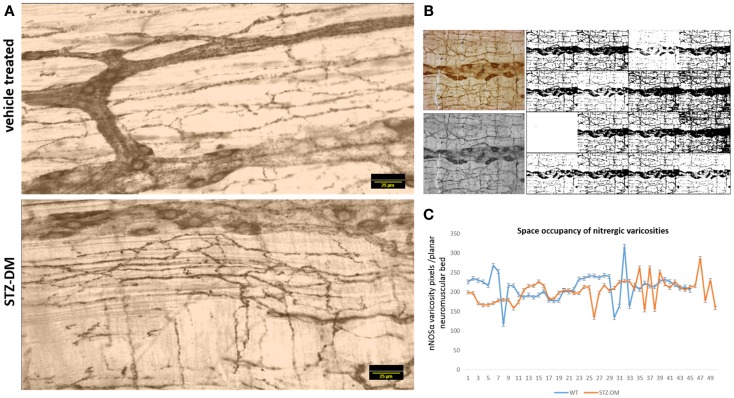
**Comparable levels of ramifications of nitrergic axonal processes and varicosities on the muscularis of vehicle treated and diabetic jejunum**. In **(A)**, the 16-bit images were pseudocolored sepia to focus on expression of the nitrergic varicosities and ignoring appearance of background during visualization. **(B)** Shows repetitive iterations of thresholding performed with NIH ImageJ. Cumulative thresholding helped subtract background and obtain accurate estimates of pixel intensities of nitrergic processes. **(C)** Shows comparative levels of space occupancy of nitrergic varicosities in wild type and diabetes, suggesting that no axonal retraction has taken place in diabetic tissues. The variance in the distribution of the axonal processes may be semiquantitatively expressed by this parameter of space occupancy derived from graph theory. Scale bar, 25 μm.

Because of the variance in the distribution patterns of nitrergic nerve processes within the muscular wall, examination of a single field of whole mount may not be accurately reflective of axonal arborization. In order to obtain quantitative evidence of intactness of arborization pattern of nitrergic axons and varicosities, we incorporated a simple application of graph theory. Essentially, we argued that a unit of muscle segment delimited on the sides by ganglion and the primary and secondary plexus constitutes a unit of muscle bed that is supplied by axonal processes extending into this region from the adjacent plexi and cell bodies. One short coming of this approach is that only a planar layer was examined, while it is possible that the ramification of the nerve fibers may be deep to the visualized tissue plane. Considering obtaining a semiquantitative estimate, only planar images were considered, ignoring the stereological aspect. In comparison to previous reports, which have shown a single section to comment on nitrergic nerve process distribution (8), we estimated the total intensity of the axonal extensions and normalized to the muscular area of distribution. The total intensity was obtained by numerous iterations of thresholding (Figure 9B), the averaging technique contributing to closely accurate estimates of pixel integrated densities. In this way, we computed the space occupancy of nitrergic processes. Because the intervaricose segments are much less stained than the varicosity regions, so it is logical to extrapolate this calculation to be an estimate of space occupancy of nitrergic varicosities. Though some diabetic sections showed a diminution in extension of nitrergic processes across muscle bed (lowest count 153 AU/μm^2^), cumulative analyses show comparable levels of the space occupancy of nitrergic varicosities (215.25 ± 0.74 vs. 209.84 ± 0.82 AU/μm^2^, *p* = 0.42, two-tailed *t* test) (Figures 9A and 9C).

### nNOSα concentrations are comparable between vehicle treated and diabetic musculomotor nerve terminals

**Figure 10:**
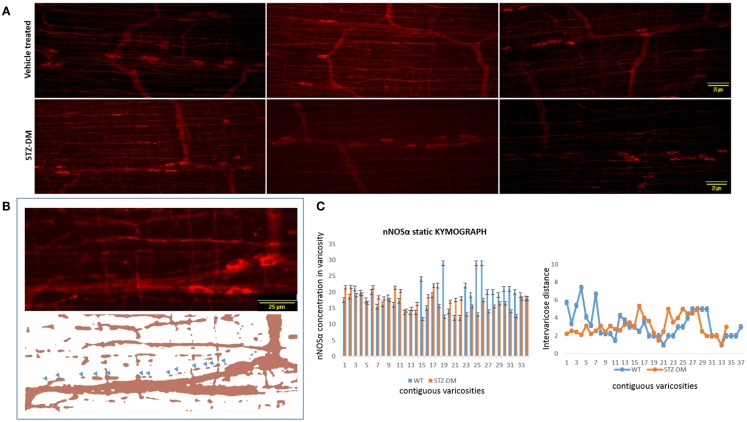
**Comparative levels of expression of nNOSα in nerve terminals of both wild type and diabetic tissues (A)**. Upper panel shows variance in appearance of nNOSα in vehicle treated and diabetes. Comparative quantitation demonstrated similar nNOSα levels within varicosities. **(B)** Fluorescent images were thresholded to digitally isolate individual nitrergic varicosities. Varicosity areas were estimated with ImageJ. nNOSα fluorescent intensities within varicosities were estimated with unaltered images. **(C)** nNOSα static kymograph shows normal axonal transport of nNOSα in diabetic jejunum to the farthest varicosities. Intervaricose distances were comparable between groups. Scale bar, 25 μm.

The preceding analyses suggest that while myosin Va expression is nearly absent in the motor nerve terminals and significantly reduced or absent in the pan-neuronal soma of the myenteric plexus from which the processes arise, nNOSα-positive nerve fibers appear normal in diabetic jejunum. Fluorescent images of nNOSα were used to examine specific nNOSα expression within the nerve terminals (Figure 10A). The images were thresholded to define the shape of individual varicosities and ease in estimation of the area of the varicosity (Figure 10B). Fluorescence intensities of nNOS expression were normalized to the areas and these values were comparable between vehicle treated and STZ-induced diabetic jejunum (18.9 ± 0.24 vs. 17.32 ± 0.15 AU/μm^2^, *p* = 0.8, two-tailed *t* test). The intervaricose distances between nitrergic varicosities were comparable between vehicle treated and STZ-induced diabetes (3.22 ± 0.27 vs. 3.08 ± 0.18 μm) (Figure 10C).

### Static kymograph shows intact nNOSα axonal transport in diabetic jejunum

Static kymograph of nNOSα axonal transport was computed (Figure 10C). Nine randomly chosen nitrergic neurons from different rats with traceable and distinctly appearing varicosities in each group were used to obtain quantitative estimate of nNOSα axonal transport. The kymograph shows that despite some reduction of nNOSα expression in few of the nerve terminals, nNOSα distribution is comparable between wild type and diabetic axonal processes, and that nNOSα reaches the farthest neuromuscular terminals in diabetic rats. This indicates that the axonal transport of nNOSα is likely not impeded in diabetes, unlike myosin Va axonal transport, which is severely impaired.

## Discussion

The results of the current study reveal that in contrast to vehicle treated rats, jejunum obtained 16 weeks after rats were injected with low dose STZ show: (a) nearly absent myosin Va expression in nerve terminals of fibers traversing the enteric neuromuscular bed, (b) variable decrease of myosin Va in cell bodies within the myenteric plexus, with near absence of myosin Va in several ganglionic neurons, and (c) some presence of myosin Va in the AIS of ganglionic cell bodies that stained for myosin Va, but near complete absence of myosin Va staining even in the initial segments of the primary plexus. In contrast, nNOSα staining in diabetic jejunum neuromuscular strips showed: (a) near intact expression of nNOSα in neuronal cell bodies, with reduction of expression in few cell bodies, (b) intact presence of nitrergic nerve fibers, with normal ramification and arborization patterns of nitrergic nerve fibers, normal density of nitrergic nerve fibers and terminals in comparison with untreated animals and normal concentration of nNOSα within a majority of nerve terminals and intact axonal transport of nNOSα to distant nerve terminals.

This report has examined the distribution of nNOSα quantitatively in diabetic enteric nerve terminals. While there was reduction of nNOSα expression in some cell bodies, as well as in some nerve terminals, the images, as well as the quantitative analyses show that there is a wide variance of expression of nNOSα both in wild type tissues, as well as after STZ treatment. Thus, mere western blots of whole gut extracts (9, 10) may not be optimal approach for revealing the dynamic changes in nNOSα expression. Cumulative analyses, in fact, reveal that there is no statistically significant reduction of nNOSα expression within the nerve terminals in STZ-treated rat jejunum. Studies have demonstrated and discussed the progressive nature of diabetic autonomic neurodegenerative changes (3, 22). The results presented in this study needs to be cautiously interpreted in the background of the single time point that has been examined.

The findings of the unaltered expression of nNOSα within the nerve terminals are sharply contrasted with the near absence of myosin Va within the neuromuscular nerve varicosities. Quantitative density plots revealed only patchy and spotty appearance of myosin Va-positive nerve terminals after STZ treatment. It may be noted that there is a wide variance of myosin Va expression in the cell bodies comprising the ganglia in diabetes. A wide range of fields have been shown to demonstrate that an entire spectrum of changes has occurred in myosin Va expression within the neuronal soma after diabetes induction. In some cell bodies, the myosin Va expression appears comparable to those of untreated animals. However, most neurons show moderate to severe reduction of expression of myosin Va. Interestingly, even when myosin Va expression is visualized within the neuronal soma, it is very difficult to observe any amount of expression of myosin Va within the processes emanating from the Dogiel type 1 and 2 neurons. One possibility may be that the primary processes may be in a different plane than the one visualized and imaged. However, close visualization failed to show myosin Va in the axonal extensions. There may be two plausible reasons, which may have contributed to this state: (a) the low synthesis of myosin Va may have produced inadequate levels of the protein so that no substantial protein was available for transport to the axon, (b) this observation may also result from alterations in the protein quality, e.g., glycosylation of myosin Va may have transformed the protein to an effete nature, precluding its normal function and transit to the AIS. Though myosin Va may be normally partially glycosylated, other studies have shown that excessively glycosylated myosins have reduced ATPase activity and ability of force generation through actomyosin formation (23–25). Succinylated wheat germ agglutinin (sWGA) based assays have shown that nNOS is not glycosylated (26), thus creating a dilemma of why nNOS function is so severely affected by the hyperglycemia of diabetes. The results of the current study provide the first possible hints into the molecular pathophysiology of how nitrergic dysfunction may occur in diabetic nerve terminals due to deficits of molecular motors.

The near complete absence of myosin Va within the proximal portions of neuronal extensions may also have resulted from defects in spectrin, which may form an unusual barrier at the axon hillock and prevented protein migration (27). This possibility is unlikely, since the results suggest that transport of other soluble proteins like that of nNOSα has not been impeded. Yet another possibility may be the result of defect of an allosteric partner for myosin Va that participates in axonal transport of myosin Va. Some preliminary evidence has been provided that neurofilament cooperatively participates in axonal transfer of myosin Va (28). It has been reported that neurofilament levels may be affected in pre-diabetic condition (29) and may decrease very early in diabetes (30). Decreases in neurofilaments have been reported in myenteric neuronal processes in high-fat diet induced diabesity (31). Earlier, it has also been demonstrated that despite the normal presence of neurofilaments in the soma and axons of myenteric neurons, a RT97 specific phosphorylated neurofilament epitope was deficient in diabetic rat ileum, and this was reversed when strips were incubated in insulin for 2 h (32). Alterations of neurofilaments may have potentially contributed to the total stasis of myosin Va within the nerve cell bodies seen in the current study. Electron micrographic examinations of neuronal processes within stomach tissues obtained from patients with diabetic gastroparesis have shown derangement of orientation of neurofilaments (33). Whether such spatial alterations of neurofilaments contribute to pathophysiology of loss of axonal transport of myosin Va in diabetic myenteric plexi remains to be examined.

The current investigation provides insights into axonal transport of nNOSα and some of the early changes seen in diabetic nerve cell bodies and processes. Overall analyses show that nNOSα is present in the farthest varicosity away from the cell body, in comparable concentrations to those seen in untreated animals. The arborization patterns remain unaltered and the numbers of nerve terminals on each process are comparable between untreated and diabetic samples, indicating that there is no neurite retraction. Earlier studies have reported loss of nitrergic neuronal cell bodies even in the early phase of STZ-induced diabetes (16, 31, 34–36), though not all studies examining at similar time points (8–16 weeks) after STZ treatment found loss of myenteric neurons (8, 37). Despite probable neuronal loss, the current study demonstrated intactness of spatial arborization of nitrergic nerve terminals in the jejunum after 16 weeks of diabetes induction. Our results are in coherence with earlier observations of intactness of nNOS expression made in stomach biopsies obtained from patient samples of diabetic gastroparesis (38). Within the cell bodies, we have demonstrated comparable levels of expression of nNOSα between untreated and diabetes. The montage images of the cell bodies show the wide variability of expression, reiterating that mere protein estimates after blotting whole tissue extracts will provide inadequate insights into the state of expression of nNOSα in untreated and diabetic tissues. The results of the current study show that density of the enteric nerve terminals serving unit area of muscularis externa is comparable between diabetes and untreated, as also the intensity of nNOSα within individual varicosities. Some diabetic tissues have shown reduced expression of nNOSα both within the cell bodies and the varicosities along the axons extending from these cell bodies, but cumulatively, there were no significant alterations. An earlier case report demonstrated loss of nitrergic innervation in a human jejunum sample obtained from a patient with long standing diabetes (39). These divergences in findings may have primarily resulted from ongoing progressive pathophysiology of nitrergic neurotransmission system.

The dissociation between unaltered expression of nNOSα and grossly reduced expression of myosin Va within enteric neuromuscular nerve terminals is the most significant finding of this study. This finding is highly significant, and provides a possible basis of explanation why repeated observations have been made of impaired pre-junctional NO synthesis, despite presence of NO synthase within nerve terminals (1, 4, 10, 11). Because intravaricosity myosin Va deficiency has been demonstrated to be critical for NO synthesis during neurotransmission (12, 14), it may be reasonable to speculate that the findings of deficiency of myosin Va within diabetic enteric nerve terminals may explain the impaired pre-junctional NO synthesis seen in diabetes. It has been reported that altered nNOS dimer/monomer ratio may be the basis of impaired NO synthesis (9), and this may have resulted from deficiency of tetrahydrobiopterin (40). Detailed biochemical analyses have revealed that tetrahydrobiopterin may contribute to dimer stabilization rather than dimer formation (41). It is possible that myosin Va deficiency results in insufficient delivery of nNOSα to the varicosity membrane, the likely function of myosin Va within varicosities (12, 42). Future studies comparing membrane-bound nNOSα dimer in wild type and diabetic tissues may resolve these issues.

The present study probably is the first analyses examining axonal transport of nNOSα and its force-generating cytoskeletal allosteric partner myosin Va and provides valuable insights into the mechanisms of axonal transport of soluble proteins. For myosin Va in the untreated group, myosin Va was visualized as discrete puncta along the nerve processes traversing the muscularis. A continuous haze of staining was only visualized in some initial segments within the primary plexus. Being a soluble protein, myosin Va likely undergoes slow transport, but may piggyback onto faster transport systems as it progresses into the axon. Importantly, there must be some mechanism for its capture at the axonal varicosities, and this possibly contributes to the punctate appearance farther along the axons. The intervaricose region here remains myosin Va-stain free, indicating that myosin Va is possibly transported as a macromolecular assembly along the axon. In diverse neuronal systems, actin and NF-L have been reported to transport myosin Va (28, 43). It remains to be examined, which allosteric protein partner(s) contribute to axonal transport of myosin Va within enteric neuronal processes. Interestingly, the axonal transport of nNOSα is not affected or only minimally affected at the 16-week time point examined in the present investigation. This may have resulted from intact transport mechanism(s) of nNOSα during the initial weeks of diabetes induction.

Most likely, nNOS-myosin Va do not form complex during transport; otherwise, nNOSα stasis would also have occurred in the neuronal cell bodies like that of myosin Va. This argument is brought in because myosin Va and nNOS may be linked by the common light chain LC8 (12, 44). However, it may be inferred from the current observations that myosin Va and nNOS are independently transported within the myenteric axons and tertiary processes. Recent evidence and debates are revealing that soluble proteins may not undergo the true form of slow axonal transport as originally reported for soluble proteins with *in vitro* experiments and radioactive pulse chase labeling (45–47). Such slow rates of about 1 mm a day are practically impossible to deliver proteins at the processes located several orders of distances away (48–51). Incipient but convincing evidence have demonstrated that soluble proteins tag on fast axonal transport systems and gets rapidly transported to distal nerve terminals (52). The major fast transport systems involve microtubules and kinesins (53). Specific nNOS–kinesin interactions have not been reported, but kinesin function deficit resulting from altered GDNF signaling in KIF26A knockout has been reported to cause Hirschsprung-like phenotype with impaired nitrergic function (54). This important issue merits future investigations. Though fast axonal transport is also affected in diabetes, this usually results from long duration of hyperglycemia. How nNOSα axonal transport is affected as a result of prolonged duration of diabetes is a relevant avenue for future testing. Incipient evidence is present regarding the existence of periaxoplasmic ribosomal plaques, mRNA, and protein translation machinery in the axons, thus likely eliminating the bottleneck necessity of synaptic and axonal protein transport (55–62). Such evidence is virtually unknown for myenteric nitrergic neurons.

Though our report is novel from the perspective of being the first report to demonstrate significant reduction of myosin Va within varicosities in the peripheral nervous system, it has been earlier reported that myosin Va is significantly reduced in neuronal cell bodies in multiple brain regions several days after STZ-induced diabetes (63). One intriguing question that posits from these observations is that if myosin Va is an important regulator of both vesicular and non-vesicular neurotransmission in both the central and peripheral nervous system, how can neuronal physiological function continue to operate, albeit at a lower level of performance, despite reduction, or absence of myosin Va. One likely reason of how neurotransmission function is sustained in the face of diminished myosin Va may be due to neuroplastic changes. For example, it has been reported that in the brain, similar diabetic conditions that result in myosin Va reduction also results in increased expression of non-muscle myosin II (NMMII) (64). NMMII plays a critical role in vesicular neurotransmitter release at the cortex of the nerve terminal. Whether NMMII plays important role in nitrergic neurotransmission merits to be investigated, though increasing argument is being placed for critical role of the cortical cytoskeleton in nitrergic neurotransmission (14). Proteomic analyses in whole gut tissue and protein identification in varicosities have shown the presence of heavy chain of NMMII (65), identified in the synaptosomes as Mg-dependent ATPase (66). Myosin light chain kinase (MLCK), the enzyme that activates NMMII, has been shown to be diminished in diabetes and this dysregulation reversed by insulin (67). However, the localization study was made in whole gut extracts and it is not known whether MLCK was down regulated in nerve terminals or smooth muscles or both.

Myosin Va expression in myenteric ganglia is much reduced in diabetic tissues in comparison to nNOSα and it seems likely that this selective reduction has taken place early on after diabetes induction. Earlier studies have actually shown increased nNOS mRNA in male diabetic tissues (9). It is possible that the transcription factor or factors that drive myosin Va genomic synthesis are exquisitely sensitive to the pathophysiological alterations resulting from STZ-induced diabetes. For example, down regulation of the snail transcription factor that binds to an E-box in the promoter region of myosin Va gene (68) may have inhibited myosin Va synthesis. It is increasingly recognized that persistent hyperglycemia can alter transcription because basal transcription operators and repressors are sensitive to intracellular glucose concentrations (36, 69, 70).

Independent reports have confirmed increased VIP-IR of nerve varicosities several weeks after STZ-induced diabetes (7, 71, 72), though studies in human diabetic tissues have not always confirmed these findings (38). While this may be related to neuroplastic changes resulting from decrease in nitrergic synthesis, the increased VIP-IR may also be related to diminution of vesicular exocytosis at the junctions (73), probably resulting from deficiency of the motor protein myosin Va. ATP exocytosis has been reported to be enhanced in diabetic enteric nerve terminals (74). This apparently confounding finding may be possible due to compensatory changes in purinergic signaling resulting from diminution in pre-junctional NO synthesis. Our observations of increased perikaryal volumes of some myosin Va neurons in diabetes may have resulted from attenuation of axonal transport. The relationship of stasis as a fundamental pathophysiology of diabetic neuropathy has long been recognized (75, 76).

Controlling hyperglycemia in the periphery through ethnopharmacologic glucosidase inhibitor obtained from *Pouteria* has the potential to reverse the loss of myosin Va in central nervous system neurons (77). This is similar to previous observations of reversal of nitrergic neuropathy by insulin treatment (8, 32). Thus, there is a potential for pharmacological reversal of myosin Va transcriptional inhibition, at least in the early phases after STZ induction of diabetes.

In summary, the results of the present study show the dissociation between presences of the key inhibitory neurotransmitter synthesizing enzyme nNOSα in the nerve terminals but absence of its local transporter myosin Va in the jejunum of rats after 16 weeks of diabetes induction with STZ. Earlier studies with dilute mice have shown that nNOSα may be present in the nerve varicosities despite deficient myosin Va (12). Surprisingly, similar observations were made in diabetic nerve terminals in the present study. STZ is uptaken by cells with the aid of GLUT2 transporter (78). STZ causes promoter methylation, gene inactivation, and cellular toxicity (78). It may be possible that alterations of myosin Va expression may have resulted from STZ toxicity of myenteric ganglia. However, this possibility is low because we did not observe global inhibition of transcription or generalized cell loss. Low dose STZ was used for the current study in accordance with previous studies, which was used as model for diabetes induction (79–82). The validity of our observations may be confirmed from different models of diabetes including genetic and lipotoxic models, examining different regions of the gastrointestinal tract and the temporal progression of disease. This significant observation of deficient myosin Va in nerve terminals may potentially explain impairment of pre-junctional NO synthesis during EFS of diabetic gut neuromuscular strips despite presence of the nitrergic synthetic enzyme nNOS.

## Author Contributions

Arun Chaudhury, conceptualized project, image analyses, drafted manuscript; Marcilio Hubner De Miranda-Neto, supervised animal experiments; Renata Virginia Fernandes Pereira, performed animal experiments; Jacqueline Nelisis Zanoni, supervised animal experiments, image acquisition and project supervision; all authors read and approved final version of the manuscript.

## Conflict of Interest Statement

The authors declare that the research was conducted in the absence of any commercial or financial relationships that could be construed as a potential conflict of interest.

## Footnotes

^1^http://imagej.nih.gov/ij

^2^http://www.imagescience.org/meijering/software/neuronj/
